# Temporal codes and recurrent timing nets for rhythmic expectancy

**DOI:** 10.3389/fncom.2026.1814579

**Published:** 2026-05-15

**Authors:** Peter Cariani, Janet M. Baker

**Affiliations:** 1 Hearing Research Center, Boston University, Boston, MA, United States; 2 Harvard Medical School, Boston, MA, United States; 3 Massachusetts Institute of Technology Media Lab, Cambridge, MA, United States

**Keywords:** neural timing networks, predictive coding, short-term memory, temporal correlations, temporal memory traces

## Abstract

This paper focuses on possible time-domain neurocomputational mechanisms for short-term anticipatory processes. Here we present a simple, signal processing functional model of how short-term rhythmic pattern expectancies could be computed on the fly using recurrent neural timing nets (RTNs). The model is inspired by Gestaltist grouping principles for repeating temporal patterns of events (beats, pulses, grooves, metrical and non-metrical patterns). Building on previous autocorrelation models of pitch, meter, and rhythm, the RTN rhythm perception model consists of temporal codes, temporal pattern memory traces circulating in delay loops, and neural delay-and-coincidence networks with dynamically-adapting spike-correlation-dependent synapses. The network tracks in parallel all event periodicities in rhythmic hierarchies. As in memory trace theories of mismatch negativity (MMN-like) neural responses, it generates simple and complex pattern expectancies and registers deviations from them. Similarities and differences of this correlation-based model with those based on oscillators and predictive coding are discussed.

## 1 Introduction

Theories of predictive coding encompass two kinds of processes, one based on top-down, experientially-acquired stored associations and another based on bottom-up shorter-term expectancies that rely on the immediate history of driving inputs (Friston, 2005). Mechanisms for associative memory depend on the contents of long-term memory stores and trained neural assemblies that can persist over lifetimes, whereas mechanisms for stimulus-driven predictions depend on echoic and short-term memory stores whose contents only persist for up to tens of seconds. This self-organization of short-term expectancies is a form of unsupervised learning in which any arbitrary repeating pattern of events creates an expectancy of its continuation. Unlike supervised learning, which leverages the structure of experienced event sequences, this process can make predictions solely on patterns of recent inputs, such that it operates on a tabula rasa that is dynamically configured by bottom-up inputs. These two processes, involving supervised learning and unsupervised, self-organization, have been integrated into unified theories of predictive coding (Friston, 2005).

Time-domain neurocomputational mechanisms for short-term anticipatory processes are possible explanations for rhythm perception. Here we present a simple, signal processing functional model of how rhythmic expectancies could be computed on the fly using temporal codes, temporal pattern memory traces circulating in delay loops, and neural delay-and-coincidence networks (neural timing nets) with dynamically adapting spike correlation-dependent synapses.

The model outlined here is intended purely as a heuristic device to show how the behavior of this mechanism might qualitatively resemble that of auditory perception, rhythmic pattern induction, and some of their neural correlates. As such, it should be regarded as an early, “demonstration-of-concept,” a.k.a. toy, model. In the spirit of auto- and cross-correlation models for pitch, binaural perception, meter, and rhythm (Licklider, 1951, 1959; Boomsliter and Creel, 1961; Cherry, 1961; Cariani, 2002; Eck, 2006; Cariani, 2011; Lyon, 2017), it is a functional neural signal processing model that relies on very general and nearly ubiquitous properties of neurons such as synaptic coincidence detection and interneural delays.

More specific groundings in biophysical processes and particular neuroanatomical structures will be necessary if it is to predict neurophysiological observables such as mismatch negativity (MMR) and N1 patterns in event-related potentials. Our main message here is that neural temporal codes and neural processing of them provide possible substrates for anticipatory predictions that can complement existing models based on neural rate-channel codes and purely connectionist neural architectures. Although these proposed temporal coding and processing schemes are, on their face, elegant in their simplicity, we hold no assumptions and make no assertions regarding their predictive optimality.

## 2 Rhythmic pattern induction

Perhaps the most important inspiration for our approach to rhythm perception comes from Gestalt psychology Gestaltist theory of perceptual organization (Wertheimer, 1923; Köhler, 1947; Wertheimer, 1958), which is a wholistic theory of relations and patterns. In perceptual psychology, since the 1980s, this body of experimentation and thought has come to be called “scene analysis” (Kubovy, 1981; Handel, 1989; Bregman, 1990; Cariani, 2004; Winkler et al., 2009a; Cariani and Micheyl, 2012; Szabo et al., 2016) the “segmentation and binding problem” (von der Malsburg, 1981/1994) or, in the context of automatic speaker separation, simply as the “cocktail party problem” (Haykin and Chen, 2005).

Rhythmic pattern induction is a perceptual phenomenon in which rhythmic pattern expectancies are generated by repeating temporal patterns of discrete events. Events here will be defined as transient stimulus discontinuities that cause abrupt, discrete perceptual changes (e.g., the onset of a musical note following silence or the abrupt change of one note to a new one). Rhythm here is used in a broad sense to refer to any temporal pattern of distinguishable, successive sensory events. The pattern may or may not be either regular or repetitive, as in the rhythm of a spoken sentence.

The power of repetition in music is widely appreciated, and most musical genres rely heavily on repeating sequences of events (Margulis, 2014). These can be temporal sequences of event timings and accents (musical rhythms), of pitches (melodies) or even timbres (tone color melodies, a.k.a. Schoenberg’s Klangfarbenmelodies, and the timbral-, phonetic- and syllabic-sequence melodies of Kurt Schwitters, Meredith Monk, and Holger Hiller). A substantial music perception literature concerns time perception (Jones, 1976; Fraisse, 1978a, 1978b), rhythmic structure (Sethares, 2007) and the formation of expectancies (Huron, 2006). Music provides strong examples of many Gestaltist grouping principles (Wertheimer, 1923; Handel, 1973; Handel, 1989; Snyder, 2000). These include proximity, similarity, good form, and good continuation (strong expectancy). When event-patterns are repeated, there is temporal pattern similarity between the patterns of each successive cycle.

The same pattern-repetition grouping process underlies perception of both metrical and non-metrical patterns. Because much of (our) music uses regular temporal frames that induce steady beats to drive our movements and expectancies, most of the research on rhythm (London, 2004; Toussaint, 2013) has focused on musical meter (beat induction and “what makes a good rhythm good”) rather than on expectations that are also produced by repeating but non-metrical rhythmic patterns. So-called metrical patterns form regular temporal grids that are subdivided by integer ratios (1/2, 1/3, 2/3, 1/4, 3/4, …, etc.), such that faster divisions reinforce the longer, overall repetition period. Faster and slower divisions share common (subharmonic) periodicities. However, repeating non-metrical patterns whose events do not fit into regular metrical grids still group together into chunks. The repetition rate of the chunks is perceived, albeit less easily than for those made up of metrical patterns.

The underlying principle is that any repeating pattern with arbitrary temporal spacings within the repetition period will produce perceived beats (a.k.a. grooves) associated with the event pattern repetition period. This repetition frequency is an “event fundamental,” and the beat pattern is perceived even if this periodicity is not present within the pattern, i.e., a “missing event fundamental” in which there is no energy at the fundamental. Rhythm production, as in finger-tapping, also fills in these “missing beats” (Tal et al., 2017). Analogously, we readily hear pitches related for harmonic complexes with “missing fundamentals.” The dominant periodicity of the whole groove pattern is only “missing” however because of the sinusoidal decomposition of Fourier analysis, but it is prominent in autocorrelation and related representations, such as RTNs and comb filterbanks (Scheirer, 1998). The question of what neural perceptual and/or motor processes might be responsible for this perceptual phenomenon is taken up in the section (7.8) on autocorrelation and oscillatory networks.

**Figure 1:**
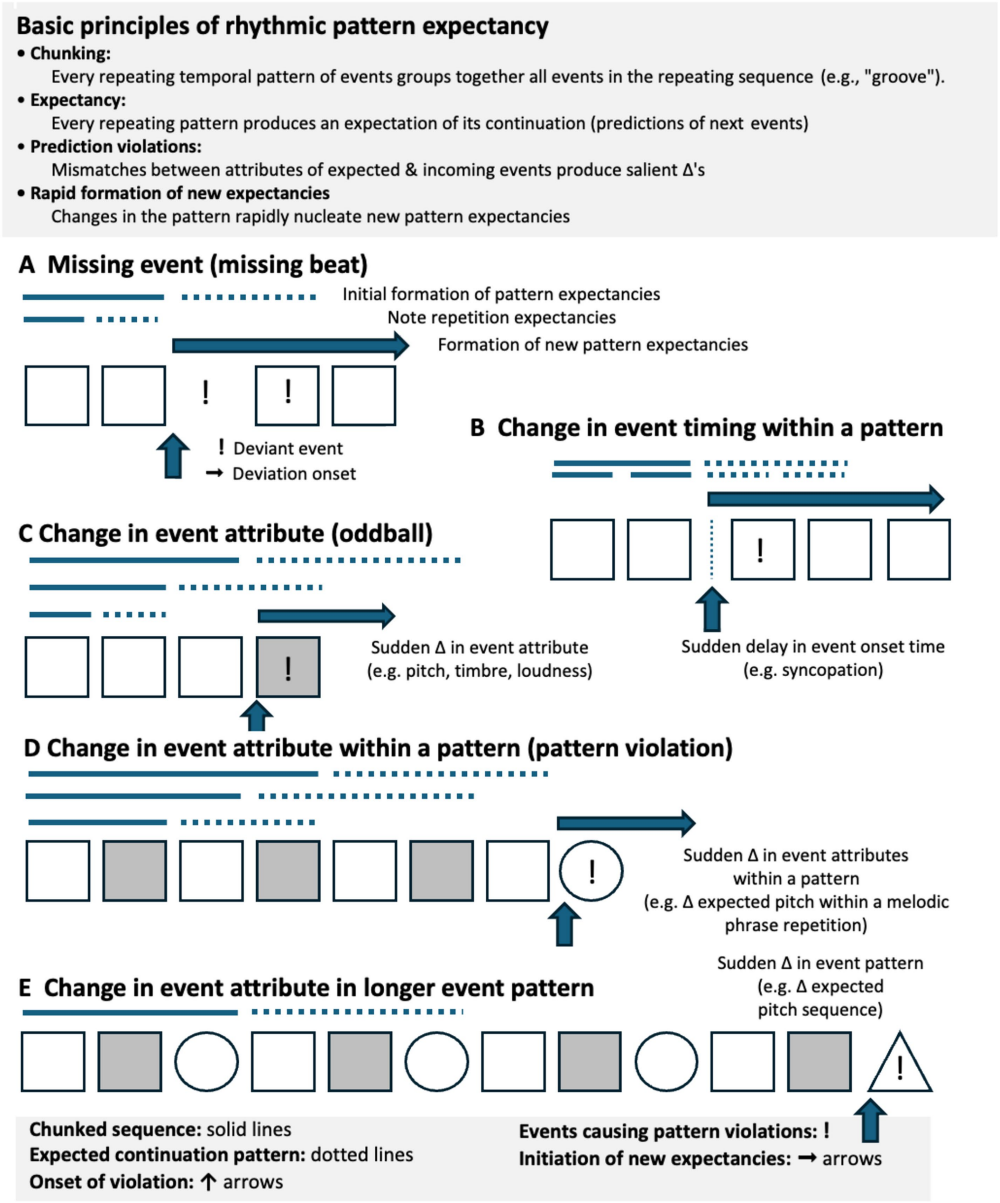
**(A)** Some basic principles of rhythmic pattern induction, grouping and expectancy. **(B–E)** Examples of rhythmic patterns, groupings of repeated patterns on multiple levels (note and group ngrams of various lengths, ones, twos, threes, etc.), and pattern deviations. Events that cause surprise, i.e. unexpected pattern deviations, have heightened salience.

Some general rules for rhythmic pattern induction, grouping, and expectation can be outlined. Musical expectations depend heavily on perceptual processes that group note-events. Figure 1 was inspired by a schematic (11.1) in Handel (1989) and the many examples of types of grouping in Snyder (2000). Rhythmic pattern induction ensues whenever repeating temporal sequences of events are presented to the senses. Events in the repeated pattern are automatically grouped together to form “chunks” or “grooves.” The repeated pattern creates an expectancy of its continuation. These short-term, repetition-induced expectancies are strong and highly independent of prior experience, such that repeating some new, never experienced pattern can easily override prior expectations.

Perception of groups of events in time exists when onsets of succeeding events fall within a given temporal integration window of ~100 ms to up to ~2–3 s. Events with inter-event intervals (IEIs) shorter than this are not differentiated, whereas streams of events with IEIs longer than this do not cohere into unified wholes. A melody played back too fast (>10 notes/s) or too slow (>2 s/note) becomes hard to recognize. The chunked sequences can extend roughly to the limits of echoic and auditory working memory, up to ~10s. The whole repeating pattern can be much, much longer, and consist of multiple chunks, but, depending on its complexity, may require presentation of a number of cycles before the repeating nature of the whole pattern is recognized.

Deviations from repeated patterns create highly salient, noticeable perceptual “pop-outs” that highlight the change between the new pattern from the previous one. For example, if the loudness, pitch, or timbre of one of the events in the pattern has suddenly changed, then the event of that change is noticed immediately following the onset time of the discontinuity. This deviancy detection is in addition to accented events within the patterns. Unexpected events that violate expectations are yet another way of further accenting musical events.

Figure 1 illustrates many of these principles. Initially a repeated temporal pattern of discrete events (solid lines) forms a group pattern that induces an expectation of its continuation (dotted lines). The expectation of continuation persists until the time point when a pattern violation occurs (vertical arrows). The violation separates the deviation from the expected pattern and increases its salience. If the violation is part of new repeating pattern, then with the repetition of the new pattern, a new set of expectations is created, and the change that caused the violation becomes part of the newly expected pattern. Once it is part of a new expected pattern, it no longer stands out by virtue of a pattern violation.

To generalize, a change in any perceptual attribute of a note sequence (event timing, event duration, pitch, timbral qualities, loudness, apparent direction), as in panels B-E, creates enhanced saliency of the “oddball” deviant event and its violation of short-term repetition-induced expectancy.

Almost invariably, contrast (surprise) causes events to be more highly accented. In musical terms, accents are events that have higher salience. The contrast can involve note-to-note changes, or on higher levels of grouping, changes from pattern expectancies. More heavily accented notes stand out from other less or unaccented notes by virtue of contrast with their immediate predecessors, e.g., being louder, longer, having sharper attacks, and being further away in tonal space.

Notes are also accented when their perceived timings (onsets) do not fit into previous metrical frames (panel B). In music, abrupt changes in note timings vis-a-vis their expected (metrical) timings create highly noticeable syncopations. Subtle deviations in the fine timing of notes vis-a-vis expected timings induced by metrical frames create readily perceived “expressive timings” that often convey emotional content. Whereas theories of musical rhythm primarily focus on metrical frames and deviations from them, the more general Gestaltist principles of grouping used here also equally apply to repeating patterns of events that do fall into metrical grids with their regular subdivisions.

## 3 Neural mismatch (MMN-like) responses

A second major source of inspiration for theories of rhythm perception comes from the phenomenon of mismatch negativity (MMN) neural responses to repeated patterns and pattern violations (Naatanen et al., 2011). The MMN behaves in a manner that strikingly resembles Gestaltist principles of perceptual organization. The N1, also called the N100, is a negative-going peak in auditory event-related potentials (AERPs) that is generated ~100 ms following acoustic contrasts that define and delineate onsets of events. In music these acoustic contrasts typically involve transient sound level changes related to note onsets (transitioning from silence to a note), but they can also be created by changes in acoustic parameters related to pitch, consonance, or timbre. Other “MMN-like” responses occur at different latencies for different types of deviations and with different neural generators have acquired specific labels (Koelsch et al., 2019; Shiramatsu and Takahashi, 2021), e.g., the middle-latency responses (MLR), stimulus-specific adaptation (SSA), and early right anterior negativity (ERAN). See (Winkler et al., 2009a) for discussion of auditory scenes, MMN-like responses, regularity analysis, and predictive coding.

These various responses are most typically observed in averaged electrical event-related potentials (ERPs) and magnetic fields (MEG MMNm) in which a standard pattern is presented with high probability with an “oddball” deviant pattern randomly interspersed with low probability. The MMN is computed by subtracting the averaged ERP waveform in response to occasional oddballs from that of the standard, more frequent event pattern.

Roughly speaking, the peak magnitude of the MMN waveform appears to reflect the degree of oddball perceptual deviancy, whereas the timing of N1 closely follows, with a characteristic delay, the timing of the emergence of the discrepancy. There has been an ongoing debate about the neural basis and functional significance of MMN-like responses. See (Picton et al., 2000; Garrido et al., 2009; Naatanen et al., 2011; Shiramatsu and Takahashi, 2021) for detailed discussions. Different, separable neural generators appear to be responsible for the different neural responses.

These neurophysiological responses are also observed when the expectancy violations are not consciously perceived, such as in some states of masked stimuli, inattention, sleep, coma and general anesthesia. MMN-like responses are also observed in human infants (Winkler et al., 2009b) and other mammals, birds, reptiles (Shiramatsu and Takahashi, 2021), and perhaps also zebrafish (Chahlova et al., 2025). Because they are physiologically observable responses that do not depend on subjective judgments or ability to report them, MMN-like measures have also been used clinically for investigating and diagnosing a host of neurological, psychiatric, and neurodevelopmental diseases (Garrido et al., 2009). A literature search on PubMed readily finds studies relating observed MMNs to depression, schizophrenia, autism, dyslexia, speech disorders, various cognitive impairments, Down’s Syndrome, Parkinson’s Disease, Huntington’s Disease, Alzheimer’s Disease, epilepsy, comatose and minimally-conscious states, as well as hearing loss, auditory memory, and other auditory dysfunctions.

MMNs are produced for violations of surprisingly complex event patterns, with pattern lengths of arbitrary complexity up to the limits of echoic and auditory working memory (Naatanen et al., 2011; Naatanen et al., 2012; Barascud et al., 2016). When recently presented temporal sequential structure is predictive of what comes next in sensory inputs, then MMN reflects that structure. In the absence of repeating structure, expectations as assessed through MMN magnitudes revert to the respective probabilities of constituent events. This behavior lends itself to statistical inference models of prediction (Friston, 2005; Lieder et al., 2013). These models have the virtue of combining expectations based on previous experience with those based on more recent sensory data.

Although the RTN model presented below qualitatively replicates much of the behavior of MMN-like measures to pattern violations, and while it relies on neurally-inspired and physiologically-plausible mechanisms, we emphasize that this simple model is not grounded in specific neural populations and therefore no claims are made for it as a model for observed MMN-like responses. Similarly, it would be well beyond the scope of this basic science commentary to speculate on its possible clinical (translational) relevance. However, the RTN model does have novel heuristic value in that it proposes that circulating temporal patterns of spikes can themselves act as temporal memory traces. These can serve directly as anticipatory, short-time predictive models for repeating patterns. The temporal nature of the neural representations that subserve rhythmic pattern perception and expectations is made explicit in this model.

## 4 Temporal coding of rhythm in the RTN model

MMNs are generated for subtle and not-so-subtle violations of temporal expectancy, as in expressive timings and syncopated rhythms in music. All neuropsychological models of rhythm perception, be they ultimately based on oscillators, clocks, interval-tuned feature detectors, time-delay networks, or timing nets, begin with time patterns of event onsets. It is well-known that in response to these onsets, neurons at all levels of the auditory system produce precisely timed spikes. At the level of the auditory cortex, jitters of first-spikes associated with an onset are on the order of hundreds of microseconds, comparable to those observed in the auditory nerve (Phillips, 1993; Heil and Irvine, 1997).

The temporal patternings of these spikes provide a direct, stimulus-locked, precise temporal representation of the rhythmic pattern. In our view, spike timings locked to event onsets is the likely basis for MMN and N1 responses that are generated when event timings are suddenly shifted. Rhythm is thus temporally coded all the way from cochlea to auditory cortex and beyond. Rhythms are not limited to audition, such that there can be rhythms perceived in patterns of flashing lights (Ando, 2009) and tactile stimulations, as well as proprioceptive and vestibular responses to movements (Todd and Lee, 2015). Rhythmic patterns can also be remembered and imagined. Rhythm can therefore be regarded as a set of supramodal attributes that includes tempo, rhythmic pattern, accent (salience) structure, and regularity. Salience and immersion are heightened when multiple modalities convey correlated rhythmic patterns, as is often the case when playing music, dancing to music, experiencing live music or even imagining it. Although we restrict ourselves here to auditory-mediated rhythms, the short-term temporal pattern expectation mechanisms likely generalize to rhythms in other modalities as well.

In addition to encoding rhythmic patterns involving coarse timescales of events involving fractions of seconds to seconds, there is also evidence for temporal coding of other auditory perceptual attributes for each event (Cariani, 1999; Cariani, 2019). These attributes include event duration, pitch, consonance, timbre (spectral brightness and shape, attack/decay onset dynamics, envelope regularity), loudness, and location. Evidence for temporal coding of sensory information exists in nearly every modality, to a much greater extent than is commonly supposed (Cariani and Baker, 2025).

We have previously discussed how temporal pattern and spike latency codes might enable multiplexing of spike patterns encoding multiple attributes of individual events (Cariani and Baker, 2022; Baker and Cariani, 2025).

**Figure 2:**
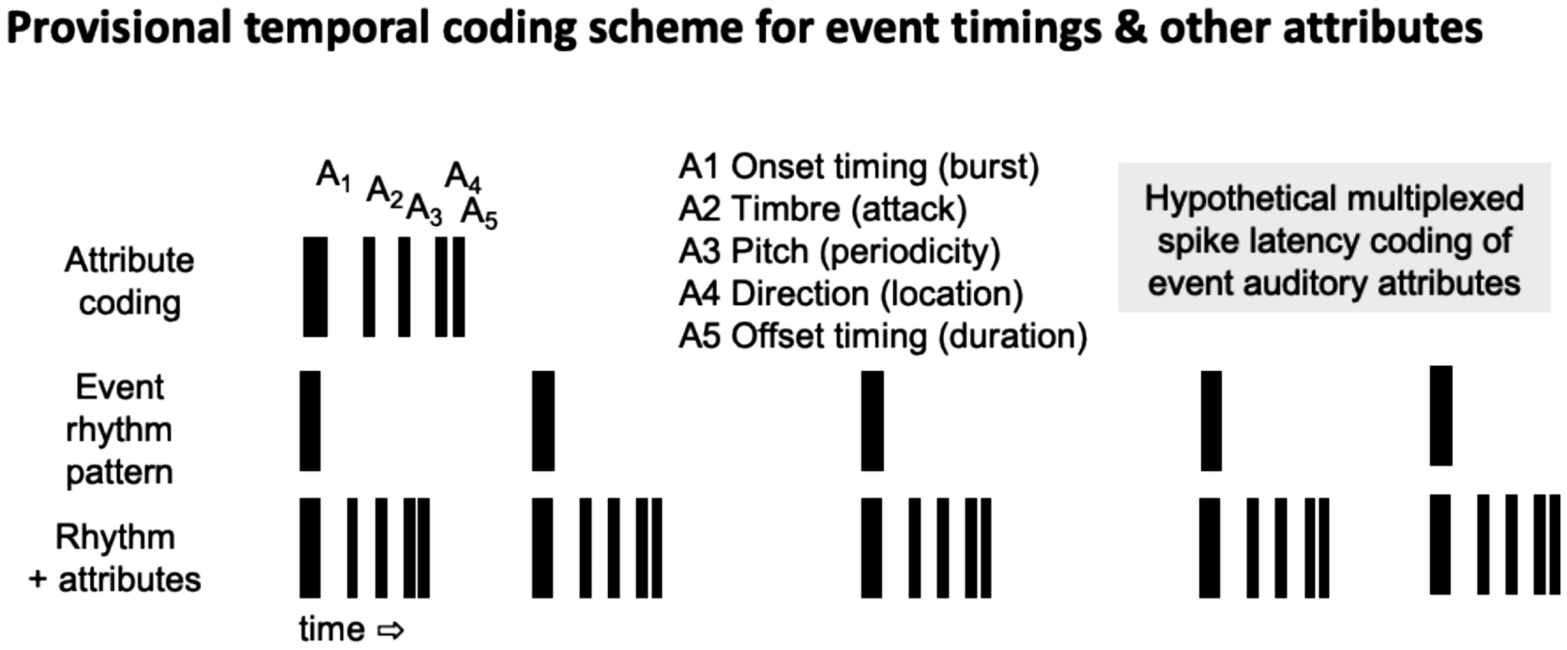
Hypothetical temporal coding scheme for representing patterns of events with different attributes. A burst pattern encodes the event onset time. Different patterns of pulse latencies relative to the onset-related burst encode other event attributes such as duration, loudness, pitch, timbre, and location in auditory space.

Figure 2 shows a hypothetical time-division multiplexing scheme based on spike latencies relative to event onsets. In such a coding framework, spikes related to event onsets and rhythmic patterns coexist with attribute-specific spike latencies. If any of these attribute-specific fine timing patterns deviate from corresponding events that have been previously presented in a repeating pattern, then the mismatch of fine timing patterns would be expected to produce an MMN and N1 with different specific latencies. More generally, if event-attributes are coded by simple or complex temporal patterns rather than different spike latencies, then changes in the representations of event-attributes will similarly produce mismatches.

Unfortunately, at cortical levels where MMNs and N1s are thought to be generated, the neural coding of the specific attributes involved is still largely an open problem. Accordingly, most existing models for rhythmic expectations and their physiological correlates sidestep the problem of the cortical representation of rhythm by simply ignoring it. When neural coding assumptions are explicitly made about sensory information and short-term memory stores, they almost always involve rate-channel codes (which neurons fire at what rates) and population-level neural mass models (Friston, 2005; Wacongne et al., 2012), rather than considering temporal coding alternatives.

## 5 Recurrent neural timing nets (RTNs)

However, as an alternative, such anticipatory mechanisms may be explicable in terms of temporal coding of sensory inputs, temporal pattern memory traces, and time-domain neural signal processing architectures. Neural timing nets are temporal correlation networks that consist of arrays of coincidence detectors and delay paths, both feedforward and recurrent (Cariani, 2001a). Figure 1 illustrates the organization of a simple recurrent timing network (RTN).

Structurally, neural timing nets can be regarded as feedforward and recurrent networks consisting of synfire chains. Synfire chains are networks of spiking coincidence-detection neuronal elements interconnected by specific time delays (Abeles, 1990; Abeles, 2003; Abeles, 2009). However, timing nets are functionally distinct from synfire chains in that their inputs and outputs are temporally-coded, i.e., information is carried in the temporal correlation structure of spiking patterns within and across neurons, rather than in channel-coded patterns of which particular neurons produce spikes.

Although, for simplicity’s sake and interpretability, RTN models have been constructed as arrays of monosynaptic loops to effect autocorrelation-like operations, in biological neural networks, we assume instead that there are immense numbers of multisynaptic delay paths available that can carry out various “delay-path computations” and correlation-based operations.

One can regard the circulating pulse patterns in recurrent timing networks as anticipatory temporal memory traces (Cariani, 2017) in which each delay loop/path is making a prediction of what the next input will be. The Russian psychologist Popov called this process of assimilating external rhythms a “cyclochronism” – “of reproducing activations in the order as that which they were originally aroused by the corresponding stimuli.” ((Fraisse, 1978a) quoting (Popov, 1950)). In conditioning experiments rhythmic stimulation produces persistent “assimilation” of rhythms in individual neurons (Morrell, 1967; Bogdanov and Galashina, 2012). This phenomenon bears much more attention than it has received.

Temporal codes and timing networks can implement auditory Gestaltist grouping and separation processes. Recurrent timing nets (RTNs) consisting of sets of delay loops and correlation-facilitated coincidence detectors were originally proposed to handle rhythmic induction in music (Cariani, 2002) and to separate concurrent voices and instruments with different fundamental frequencies (Cariani, 2004). When the fundamentals (F0s) of two voices are similar (∆F0 < 10%), their voice pitches perceptually fuse. Temporal patterns reflecting their mixed, composite waveform circulates in those delay loops with recurrence times that match the F0 period (1/F0) and its integer multiples (n/F0). When the F0s are different, the individual waveforms of each of the two complexes build up and separate into delay loops corresponding to each F0 period.

RTNs process rhythmic patterns in a similar manner. Here, for simplicity, the principles are illustrated with temporal patterns of binary pulses (0, 1), although half-wave rectified waveforms and different processing rules have also been used in past implementations (Cariani, 2001a; Cariani, 2004).

**Figure 3:**
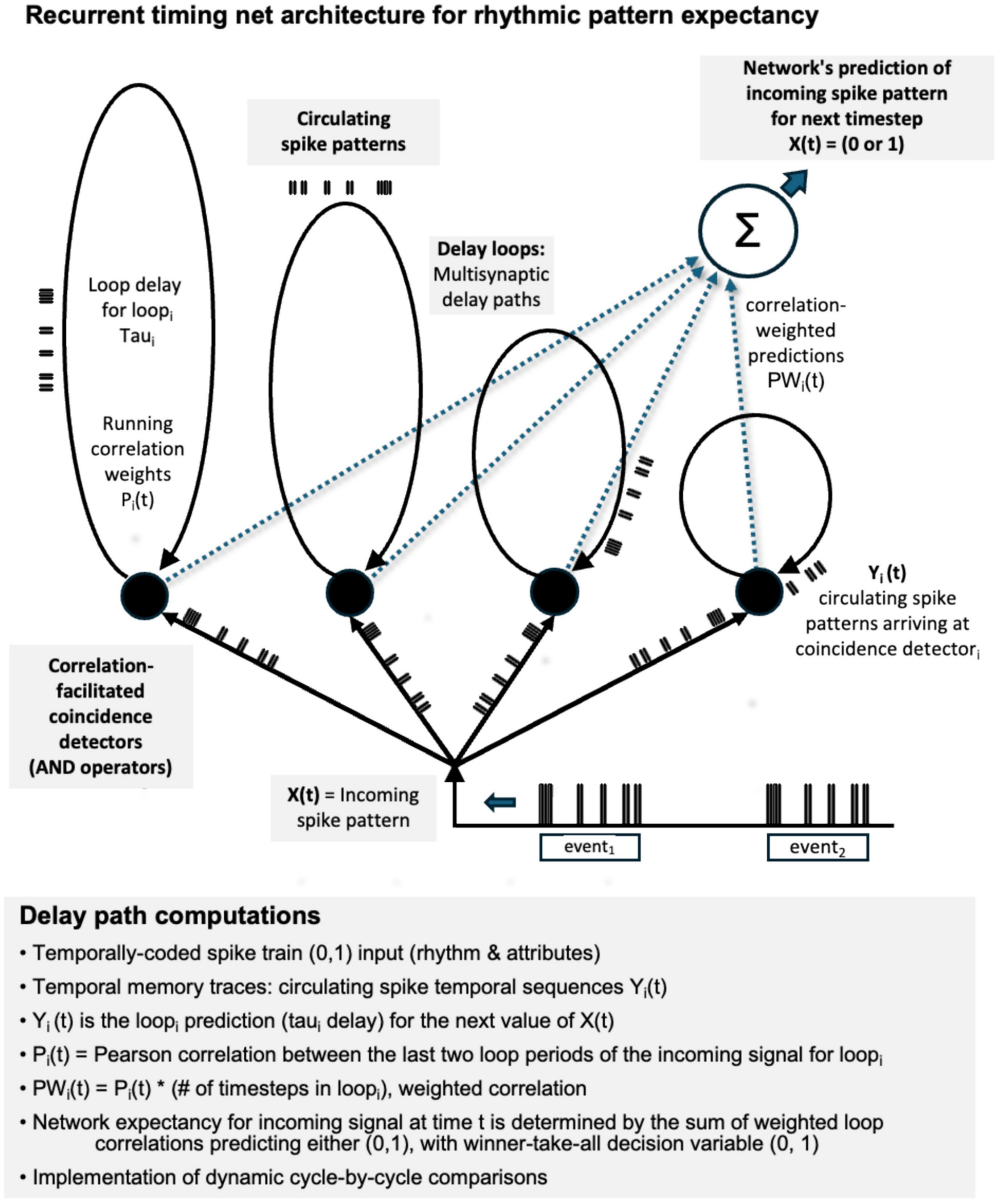
Structure of the recurrent timing net (RTN) correlation-based adaptive predictive model.

Figure 3 outlines the signal structure and signal processing for the current RTN model. Incoming pulse patterns entering the coincidence array at time t are fed into all coincidence elements and their associated delay loops. Then the pulse sequences circulate within each loop i for a given loop delay time tau _i_, arriving back at the coincidence element_i_ at time t + *tau*_i_. Each delay loop predicts that the value of the next input will be the same as the arriving, circulating signal, Y_i_, either 0 or 1. The prediction of the whole network, discussed in more detail below, is based on the weighted correlations of the delay loops predicting either a 0 (no pulse) or a 1 (pulse). Pseudocode for the computations is given in the Appendix.

The inputs and outputs of RTNs consist of temporally-coded pulse trains whose temporal patternings encode stimulus attributes. In the auditory domain, these perceptual attributes include event timings (onsets, offsets, durations) and as well as other event-attributes (e.g., pitch, timbre, loudness). Here waveforms consisting of temporally-patterns pulse sequences enter an array of recurrent delay paths with different loop delays. The pulse sequences circulate in the loops, playing the role of temporal memory traces. Each loop with its characteristic delay creates its own rhythmic expectancies.

## 6 Characteristics of RTN network behavior

The RTNs behave in a manner that appears similar to rhythm perception: grouping repeating sequences into temporal chunks, creating predictive expectancies and registering their violations. Simple isochronous rhythms build up in delay loops that correspond to the repetition period of event onsets. Complex rhythms consisting of repeating sequences of multiple event types having different attributes also build up in delay loops with recurrence times equal to the repetition period and its multiples (see below). In addition, recurring subpatterns in the sequence separate and build up in loops with their corresponding subpattern durations.

Although, as with synfire chains and cycles, neural timing nets depend on pulse-coded inputs, interneural delays and coincidence detectors, unlike synfire networks, they operate on the assumption that the inputs and outputs are both temporally-coded, i.e., that the relevant information that is processed lies in spike temporal correlation patterns and not in which particular neural elements are activated. In signal processing terms, these networks can be regarded as arrays of comb filters, cf. (Scheirer, 1998), that separate out constituent time-series signals on the basis of fundamental frequencies (F0s) and rhythmic pulse-pattern signals on the basis of repetition frequency (event-F0s). They are autocorrelation-like predictive processes based on temporal correlations amongst spikes c.f. the correlative perception theory of Tanguiane (1993) and the correlation theory of brain function (von der Malsburg, 1994). In this respect, the operations resemble linear predictive coding (LPC) and autoregression, albeit on longer timescales than previously applied in artificial speech recognition (Makhoul, 1975; Markel and Gray, 1976).

Spike patterns reverberating in recurrent delay paths (delay loops) can also be regarded as pattern oscillators in which an oscillator produces a time pattern of multiple pulses. The description of their behavior includes their recurrence time, the circulating pulse pattern in the loop, and the current phase of the pulse pattern cycle. The image of the pulse pattern circulating in a loop in Figure 3 resembles the necklace notation for musical rhythms of 13th century Bagdadi theoretician Safi al-Din al-Urmawi from his *Book of Cycles* and similar circular representations of claves (Sethares, 2007; Toussaint, 2013).

In essence the networks are doing a cycle-by-cycle comparison on repeating temporal patterns in their inputs. With appropriate sets of delays, they can separate concurrent voices/notes based on different pitch periodicities (F0s) and can separate multiple rhythmic patterns (polyrhythms) with different repetition frequencies (event-F0s) (Brown, 1993). They also can function as anticipatory predictors of future occurrences of events in their inputs.

Most of our previous applications of timing nets have involved separation of multiple complex harmonic sounds with different fundamentals (Cariani, 2001a; Cariani, 2004). Despite their different time scales, there are pervasive parallels between rhythm perception and musical pitch perception (Boomsliter and Creel, 1961). Musical pitch involves waveform repetition periods (fundamental periods, 1/F0s) of ~250 μs to ~50 ms, whereas rhythm involves event-sequence repetition periods of 100 ms up to a few seconds. Infra-pitch perception is intermediate between these two regimes (~10–20 Hz). Both pitch and rhythm involve repetitive time patterns and the grouping of the repeating pattern at the repetition period. For both, abrupt changes from cycle to cycle cause the differences to pop-out from the rest of the pattern. For pitch, these can involve changes in phase or magnitude of one harmonic in a harmonic complex. For rhythm these can involve changes in the timing of events in the sequence or any of their auditory perceptual attributes (timing, duration, pitch, timbre, loudness, location).

When the fundamentals of two concurrent musical notes are the same, the respective harmonic complexes fuse together such that one F0-pitch is heard. When the fundamentals are different, but harmonically related (e.g., 3:2, 4:3, 5:3), with common subharmonics, there is an intermediate degree of fusion in which both the fundamental of the dyad and the individual notes can be perceived. When fundamentals are not harmonically related (e.g., tritone = √2) and separated by more than a semitone (> 6%), two F0-pitches are readily heard and their respective timbral attributes can be better distinguished (as with recognition of the single-vowel constituents of “double vowels”).

When two harmonic complexes (e.g., vowels, musical notes) with different, unrelated F0’s are presented to recurrent timing nets, the temporal patterns of the two separate into different delay loops. When a repeating pattern is presented to the network, those patterns circulating in loops having the same recurrence time as the incoming, presented pattern have maximal correlation with it. The kinds of signal processing that work for separating out voices with different fundamentals (F0-pitches) readily apply to rhythm perception.

Here we outline a simple model of recurrent timing networks that dynamically assigns facilitates or suppresses the signals circulating in specific delay loops. One can regard these circulating pulse patterns as temporal memory traces in which each delay loop is making a prediction of what the next input will be. Biological brains, for the most part, consist of recurrent, re-entrant networks of time delays between neurons (delay loops and multisynaptic delay paths). The characteristic irregular firing behavior of cortical pyramidal cells strongly suggests that productions of spikes are mainly triggered by multiple coincidences amongst relatively few synaptic inputs rather than integrations over many hundreds to thousands of inputs (Abeles, 1982). If so, then spike synchronizations and interneural delay paths are critical for information processing at cortical levels (Abeles et al., 1994).

The degree to which the circulating signal in a given delay loop has correlated with the incoming signal over its recent history determines its degree of facilitation/suppression and its relative amplitude vis-a-vis other loops. The adaptive, dynamic correlation-based signal weighting mechanism in this model is qualitatively similar to spike timing dependent plasticity (STDP), albeit extended to different, shorter and longer, timescales.

The specific processes underlying neuronal coincidence detections, short-term synaptic facilitations (STDP)(Feldman, 2012), and responses to periodic stimuli can be complex and are incompletely understood. Among others, these processes may involve relative timings of excitatory and inhibitory inputs, the dynamics of calcium fluxes, AMPA and NMDA receptors (Bermudez Contreras et al., 2013; Morris, 2013), membrane recovery time-courses (Stockbridge, 1988), glial-neuronal and glial-glial interactions (Araque et al., 2014; Perea et al., 2014; Andrade-Talavera et al., 2023; Nowacka et al., 2025), and mechanisms underpinning neurally-assimilated rhythms (Morrell, 1967; Bogdanov and Galashina, 1999).

Implementation of these kinds of timing networks in real brains would require multi-synaptic delay paths through synfire chains and self-sustaining synfire cycles (Abeles, 2009). How this would be achieved with realistic biophysical processes remains to be worked out.

## 7 Basic structure and behavior of the current RTN model

In its current form (Figure 3), the RTN model for rhythm computes the Pearson correlation coefficient between the last two periods of each delay loop. The correlation values for each loop are weighted according to the duration of the loop (# timesteps). The prediction of each loop_i_ with delay tau_i_ is the current value of the circulating signal Y_i_(t), which is the value of the incoming signal X(t) tau_i_ timesteps ago, i.e., Y_i_(t) = X(t-tau_i_). At any given time, some loops will predict that X(t) = 0, while others will predict X(t) = 1. The sum of weighted correlations for loops predicting either 0 or 1 are compared, with the greater value determining the network prediction at that time (winner-take-all). Prediction error (degree of divergence between network expectation and the incoming signal) is quantified by the difference of the two sums when an incorrect decision is made. The horizontal markings in the bars indicate the normalized magnitude of this error. A pseudocode description of the algorithm is given in the Appendix.

The prediction of the entire recurrent timing network is then roughly the correlation weighted sum of all the circulating signals in the network. It should be emphasized that the model for rhythm is still in a rudimentary stage of development. Alternative specific rules for computing the predictive efficacies of running, fast-adapting delay paths, such as weighting the recency of correlations within each delay loop, are under consideration. In addition, the outputs of the loops could be cross-correlated with each other and/or added directly to the incoming signal.

**Figure 4:**
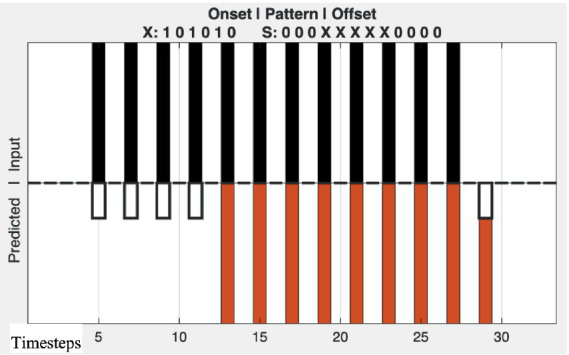
Behavior of the RTN for an isochronous sequence of events. The input event pattern is shown on top (each black bar represents one event, without coding of attributes). Silent segments preceding, during, and the rhythmic pattern are indicated by the horizontal lines in the middle of the plot. The predictions of the RTN are shown on the bottom half (red bars). Error magnitudes are indicated by the height of the open bars.

Figure 4 shows the behavior of the network in response to an isochronous rhythm. The initial silent period sets up an expectation of its continuation and initial expectancy violations for a few timesteps until the delay channels become populated with the isochronous pulse pattern. The network continues to track the isochronous rhythm until the pulses end with silence, creating another mismatch between delayed circulating pulse patterns and the incoming silence.

**Figure 5:**
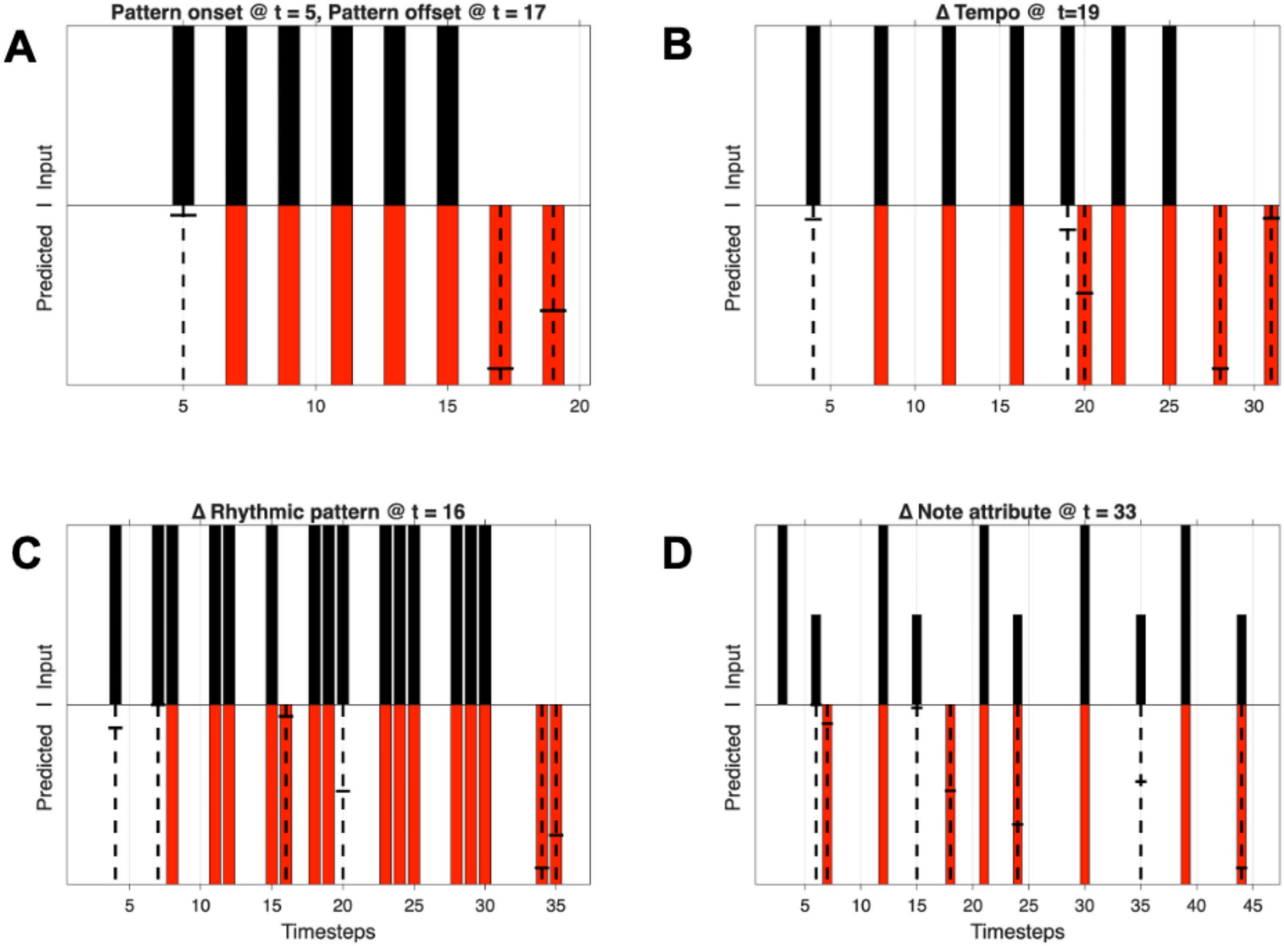
Behavior of the adaptive RTN model. Top plot sections: black bars show stimulus events (0, 1). Bottom sections: Red bars show network expectancies for events (0, 1), dashed vertical lines in bottom show expectancy violations (network expectancy ≠ incoming signal). Short horizontal lines show relative predictive error magnitudes normalized for each plot. **(A)** Network response to onset and offset of an isochronous rhythmic pattern. **(B)** Response to an abrupt change in tempo. **(C)** Response to a change in rhythmic pattern. **(D)** Response to a latency-coded change in a note event's attribute value such as loudness, pitch, or timbre ("Δ Note attribute"). Longer bars: timings of spiking events associated with event onsets, shorter bars: timings, latencies re: onsets, that encode some stimulus attribute.

Figure 5 shows model predictions for several different types of rhythmic patterns and their violations.

Several general observations about the behavior of the network can be made. Repeating pulse patterns circulate, self-reinforce, and segregate in delay paths whose recurrence times equal event periodicities in the pattern. This includes the repetition period or its multiples, even if the “event-F0” or particular beat rates are “missing” in Fourier descriptions. Those delay loops that match event periodicities are invariably the ones with the highest running autocorrelations and therefore are weighted most heavily in the model. It should be noted that the multiples of event repetition periods are resonating to subharmonics of event periodicities. The “beat frequency” (or event-F0 or frequency of the metrical “pulse”) that is followed can correspond to any of these subharmonics. The network rapidly adapts to new patterns of variable lengths.

Such architectures produce behaviors that qualitatively resemble groupings and expectancies in rhythm perception. They produce rhythmic pattern expectancies for any repeating pattern, simple or complex. Simple, isochronous rhythms create the strongest expectancies. They can handle temporal sequences of arbitrary length (cf. variable-length ngrams of symbols and words), up to the limits of the delays that are available. As observed in human event-related potentials (Snyder and Large, 2005; Zanto et al., 2006; Winkler et al., 2009b; Tal et al., 2017), when beats are missing within an isochronous sequence, there is an expectation produced by the RTN that fills them in. When a discontinuity or pattern violation appears, differences between the pattern of incoming events and those predicted by the network appear. As with finger-tapping and musicians adapting to changes in tempo, within two periods the RTNs adapt to recurring temporal patterns of events regardless of the repetition periods of the patterns.

## 8 Relations to other theories of rhythm perception

Some similarities and differences between theories of rhythm perception are worth considering. Where does a temporal coding theory of rhythm fit in vis-a-vis other general approaches? How is our RTN model related to models based on oscillatory processes and endogenous rhythms and resonances? What is novel about the model and what is not?

### 8.1 The landscape of theories of rhythm

First, it is necessary to survey the various theories of rhythm and rhythmic expectancy. Although no one source covers all theories, there are some excellent starting points (Handel, 1989; Bigand, 1993; Snyder, 2000; London, 2004; Sethares, 2007; Honing, 2013; Large et al., 2015; Jones, 2016; Honing and Bouwer, 2019; Koelsch et al., 2019; Large and Kim, 2019; Large et al., 2023).

Theories of rhythm cover a wide gamut of different types and concerns. Rhythm models can be distinguished and grouped in terms of their guiding paradigms. Although the categories listed below are largely separable by their purposes (what aspects of rhythm they seek to explain) and contents (in what terms do they explain these). The categories are not mutually-exclusive such that a given approach can fall under two or more of these types. These include perception theories, cognition theories, motor or rhythm production theories, musicological theories, signal processing theories, neurocomputational theories, statistical theories, and predictive coding theories.

Theories of rhythm and time perception are concerned with the nature and structure of mental representations of rhythm and time (Fraisse, 1978a, 1978b). Perception of temporal patterns seek to explain under what conditions and how rhythmic patterns are detected, discriminated, compared, and recognized. Their focus includes Gestaltist grouping phenomena, rhythmic pattern invariances. and draw on the methods of experimental psychology and psychoacoustics. Internal clock models for rhythm perception (Essens and Povel, 1985) overlap with cognitive models.

Motor theories of rhythm focus on rhythm production. These include motor theories of rhythm perception that describe rhythm-related behaviors, such as finger tapping and beat following, in terms of oscillators and dynamical systems.

Cognitive theories of rhythm model rhythm in terms of syntactic, hierarchical structures that are generated from bottom-up Gestaltist grouping rules, top-down schemas and transformations. Modeling of rhythm focuses almost exclusively on musical meter.

Coming from music theory, systematic musicology, and ethnomusicology, descriptive, prescriptive, and comparative treatments of musical rhythm describe the various rhythmic patterns used in music and their role in music analysis, composition, and performance.

Signal processing theories of rhythm analyze temporal patterns of events in terms of signals, analytical representations, and signal transformations that resemble the structure of rhythm perception (Sethares, 2007; Tanguiane, 1993).

Neurocomputational theories attempt to describe rhythm perception and/or production in terms of neural mechanisms. These include neural timing nets, dynamical attending oscillator models, tuned neural modulation detector models, neural predictive coding models (Wacongne et al., 2011; Wacongne et al., 2012).

Statistical theories posit that rhythm perception is based on the long- and short-term statistics of event-patterns (Temperley, 2007).

Predictive coding theories consider rhythm perception in terms of anticipatory, predictive processes (Temperley, 2001; Friston, 2005, 2010; Wacongne et al., 2011; Wacongne et al., 2012; Clark, 2013; Koelsch et al., 2019).

### 8.2 Rhythm as supramodal temporal pattern perception

Perception-based models approach rhythm as a form of auditory, vibrotactile, vestibular or proprioceptive temporal pattern perception. Rhythm perception can involve many sources in the brain, not just from one sense modality. We view rhythm as supramodal, which can include internally-generated neural time patterns, especially those originating from motor systems when rhythms of movement are being produced, and from memory when auditory rhythms or movements are being imagined (Cheng et al., 2022). No one modality is the sole, obligatory seat of rhythm.

Although primary examples come from musical rhythm, as mediated through the auditory modality, rhythm is similar in many respects to pitch perception (Boomsliter and Creel, 1961; Cariani, 2002), visual perception (flashing lights, moving gratings of multiple spatial frequency harmonics (Ando, 2009)), and vibrotactile pattern perception (Marozeau et al., 2026). All of these create percepts related to “missing” fundamentals. Training to recognize rhythmic patterns presented in one modality can be transferred to the same patterns presented in another, even in bees (Zeng et al., 2026). The vestibular system also produces spikes that are phase-locked to intense sounds as well as head movements (Todd and Cody, 2000; Todd and Lee, 2015), such that there are temporal representations of rhythms that complement those mediated by the auditory system. This RTN model focuses on rhythm perception rather than production.

### 8.3 Motor theories of rhythm perception

Motor-centric, rhythm production theories see rhythm primarily in terms of motor productions (what rhythmic patterns can be produced, as evidenced by finger-tapping and metrical beat following). Extreme motor theories of perception, such as strong motor theories of speech, assert that there is no perception without translation into or involvement of motor gestures and programs. Models are tested using finger-tapping and other movements related to beat-following. While this is perfectly reasonable for investigating rhythm production, it is not necessarily appropriate for studying rhythm perception. The main reason is that we perceive more rhythmic distinctions and details than we can produce. Although being a musician (especially a drummer) or a dancer increases awareness of nuances of rhythm, by far most nonmusicians and nondancers (including author PC) can perceive much greater rhythmic detail than they can produce with their bodies.

Finger-tapping to rhythmic sounds or following musical beats is reasonable if one is studying rhythm *production*, but, arguably, not the best way to assess rhythm *perception*. If a rhythmic pattern can be reproduced, then one can infer that the perceptual system has some means of sensing and representing it. However, if a rhythmic pattern cannot be reproduced, then one cannot infer that it is not perceived. Using rhythm-tapping to make inferences about rhythm perception is analogous to studying pitch perception by measuring the ability to sing a note that matches the pitch one hears.

The reliance on rhythm production has greatly distorted how we think about rhythm perception in animals. Requiring animals to tap out rhythms or follow beats has radically narrowed our estimation of what rhythmic patterns animals can perceive, discriminate, and recognize. However, the more we learn about animal rhythm perception (Zeng et al., 2026), as opposed to rhythm production or motoric beat-following, the more universal the perceptual capabilities, if not “human-like” beat-producing behaviors (Patel and Iversen, 2014), appear to be.

### 8.4 Dynamic attending theories of rhythm perception

Dynamic attending theories based on linear and nonlinear coupled oscillators come out of an enactivist, motoric, action-oriented tradition. They are inspired by examples from physics and dyanamical systems theory. These, along with predictive coding, are currently the dominant, neurocomputational theories of rhythm. The relation of the RTN model to these theories is discussed further on.

Dynamic attending models that incorporate rhythmic expectancies were first proposed by Jones (1976), Barnes and Jones (2000), and Jones (2016) and have subsequently been refined by Large and Jones (1998), Large et al. (2015), Large and Kim (2019), and Large et al. (2023). More recent versions are being called Neural Resonance Theory (NRT) (Large et al., 2023; Harding et al., 2025). Dynamic attending models are essentially motor theories of rhythm perception in that they hold that motor systems involved with rhythm production greatly influence, if not completely drive, rhythm perception.

Some history is in order. Rhythm has been conceptualized by many neuroscientists and psychologists primarily in terms of biological rhythmic movement. Repetitive movements point toward oscillatory muscle actions and the natural physical resonances of bodily limbs. The early lineage of oscillatory mechanisms for movement runs from Sherrington to Brown to von Holst, who studied rhythm in the context of repetitive animal movements (Gallistel, 1980). Early models of movement sequences relied on chaining of individual movement segments, i.e., the surface structure of events. These simple models, unlike rhythm perception, were not robust with respect to omitted and inserted events. These “serial structure” models were subsequently demolished by the arguments of Lashley (1951). It became quite clear that multi-level, “hierarchical” representations that encompassed both surface (event-to-subsequent-event sequences of time intervals) and deep structures (longer temporal groupings of events) were needed (Martin, 1972; Povel, 1981).

These multilevel metrical representational structures simultaneously include longer “beat” level periodicities, and several levels of groupings of faster periodicities nested within them. These different levels are created by groupings of events, and they are described in terms of pulse, beat, tactus, meter, groove, measure, and other terms (Cooper and Meyer, 1963; Sethares, 2007). The music literature can be confusing because their various meanings can vary considerably according to the types of music being considered. Often, it is assumed that readers already understand their musical correlates, and consequently, they are rarely clearly defined, i.e., in operational terms.

Dynamical attending theories draw on networks of oscillators that have different resonant frequences. Early models considered linear (simple, harmonic) oscillators, whereas later models deployed nonlinear oscillators and tracked their quasi-periodic trajectories through dynamical systems phase spaces. When presented with rhythmic input patterns, the variously tuned oscillators resonate with stimulus periodicities near their resonant frequencies. Oscillatory phases, frequencies and amplitudes are thereby modified in those oscillatory elements. The result is that the network realizes a multilevel hierarchy of resonance periodicities, much like the various delay loops of the RTN. As in dynamic attending theories, we would assume that the outputs of RTNs in auditory areas are further filtered by motor resonances in order to predict finger-tapping and beat-following behaviors.

Both oscillator models and timing nets depend on delays for their responses to temporally-patterned inputs. The delays can reside either in the oscillators in the form of recovery times or in conduction paths in the form of recurrence times. However, a major difference between the two types of delays and consequently, in the two types of networks, is that each RTN conduction delay loop (or multisynaptic path) carries the whole pattern, with the loops whose recurrent times match stimulus periodicities being weighted (amplified) by their recent autocorrelation history. This allows the conduction delay path to act like a “tape recorder memory.” In contrast, the amplitudes of the outputs of oscillatory elements are increased or decreased according to the proximity of their resonant frequencies with periodicities in the rhythm (they act in a manner not unlike bandpass filters). The summed output of the oscillators in the network consequently reflects the dominant periodicities in the just-presented rhythmic pattern. Its amplitude at any given time can be interpreted as the network’s prediction of what will happen next in the input.

The RTN and the oscillator networks are very similar in this respect. Both produce temporal patterns of response, i.e., both operate to produce time-series outputs (time-domain) rather than channel-coded patterns of which neurons are activated. In discussions of RTNs and our time-domain brain theory (Baker and Cariani, 2025), we use the terminology of temporal coding, rather than using dynamical systems terms such as entrainments, resonances, mode-lockings, Arnold tongues, attractors, and the like.

As in dynamic attending and neural resonance theories, the output patterns themselves (oscillations, interspike interval distributions) constitute the neural productions of the system. In our terms we regard the output patterns as the “signals of the system” (Cariani and Baker, 2022; Baker and Cariani, 2025) or as “neural representations” in its broader, neuroscience and signal-processing senses rather than its traditional narrower, symbolic cognitive science sense. Historically, theories of perception and action based on oscillators and dynamical systems developed within the context of Gibsonian, ecological psychology (Michaels and Carello, 1981; Turvey, 2018), enactivist, and behaviorist traditions that eschew cognitive science terms such as “representations.” “A neural oscillation is not a representation of a rhythm” (Large et al., 2023). They regard these terms as mentalistic, structuralist, and purely psychological constructs, as opposed to those used in physics or mathematics. However, there can be reasonable mappings, translations, and reproachments between the different sets of concepts and their terminologies (Cariani, 2001b). For example, the attractor basins of dynamical systems theories correspond to the symbols of cognitive science.

The “dynamic attending” label implies that motoric oscillatory patterns reinforce periodicities in the stimulus and/or create new ones, so as to heighten attention on those aspects of the stimulus time structure. We think of attention in terms of signal selection. This can happen in two ways: first by regulating channel gains, and second by signal-signal interactions (“signal dynamics”). Channel gains are regulated by relative facilitation (amplification) or suppression (attenuation) of neural channels that carry relevant signals. Channels can also be pre-empted or swamped by masking stimuli, e.g., a “line-busy” mechanism. Secondly this can happen by signal interactions that reinforce or destructively interfere with incoming signals. In this signal-dynamics manner, internally-generated signals can act as matched filters that can enhance or suppress selected incoming signals. For temporally-coded signals, this can be effected via cross-correlation operations. This latter type of attentional mechanism, based on common temporal patterns (correlations), is similar to the idea of enhancing neural periodicities by means of oscillatory coupling.

A further possible difference between RTNs and dynamic attending models concerns beat-tracking, as observed through finger-tapping, when there are repeating event patterns that have “missing beat fundamental” frequencies (Tal et al., 2017), i.e., there is no acoustic energy at the event-F0 (the pattern repetition rate or beat rate) in Fourier descriptions of the stimulus. Nonlinear oscillators and nonlinear multiplicative “mixing” of signals can, among a variety of many other nonlinear processes (e.g., bursting (Tal et al., 2020)), produce new frequencies in their outputs.

These new frequencies can be observed in MEG/EEG recordings that match the pattern repetition, “beat” frequency that is produced by subjects who are tapping their fingers to the rhythmic patterns. These extra frequencies related to the finger movements appear even when they are absent from the Fourier descriptions of the rhythmic acoustic pattern. Dynamic attending theory interprets these findings in terms of nonlinear interactions between neural oscillators in motor and auditory systems, with motor systems primarily having endogenous, intrinsic oscillatory modes and the auditory system primarily reflecting stimulus-driven, repetitive time structure. These findings are discussed in more depth in the section below on autocorrelation vs. oscillator networks. In addition to intrinsic oscillatory modes in motor systems, there are, of course, in brains a multitude of other brain rhythms and associated functions, with many possible implications for disease (Buzsáki and Watson, 2012).

### 8.5 Mixed sensorimotor models of rhythm perception

A mixed sensorimotor model in which rhythm perception is co-determined by auditory, vestibular, and motoric factors (“rhythm perception is a form of vestibular perception”) has been proposed by Todd and Lee (2015). In critiquing motor theories of rhythm perception, they rightly point out that rhythm perception does just fine in the absence of voluntary movements (those of us who are neither dancers nor musicians tend to agree). Future RTN models would do well to incorporate temporal patterns of vestibular inputs and motoric action alongside auditory ones and along with expectancies from long-term memory.

### 8.6 Cognitive, signal processing, neurocomputational models

Cognitive, structures-and-rules models regard rhythm in terms of multi-level hierarchically-organized “musical grammars” consisting of syntactic tree structures and transformational rules that are similar in character to Schenkerian analysis and Chomskian grammars (Lerdahl and Jackendoff, 1983; Longuet-Higgins, 1987; Temperley, 2001).

One can have neurobiology-inspired signal processing practical, engineering models and engineering-inspired neural signal processing models. RTN’s have aspects of both.

Signal-processing models focus on signals and operations on them that produce system functional states and behavior patterns that emulate those of humans and animals. A broad survey of signal processing models for rhythm can be found in Sethares (2007). Some other specific examples include (Tanguiane, 1993; Scheirer, 1998; Teki et al., 2011).

Neurocomputational models seek to ground their processing of rhythmic patterns in the structures and functions of biological neural networks. These include this RTN model, oscillator-networks, and the abovementioned neural modulation spectrum and sensori-motor models (Todd and Lee, 2015). Neurally-inspired signal processing models directed at practical, engineering applications fit naturally under this rubric (Lyon, 2017).

Neural signal processing and functional neuropsychological neurocomputational models, such as the RTN model here, attempt to explain and predict psychological functions on the basis of neural activity patterns (e.g., observed or simulated spike trains, collective ensemble and population responses). These neural signal processing models are mid-level descriptions that lie between high-level patterns-and-rules approaches and low-level, physics-inspired, high-dimensional dynamical systems approaches (Cariani, 2001b).

### 8.7 Difficulties of comparing the theories

Direct comparisons can be difficult because the various theories:

- Seek to explain different aspects of rhythm (explananda, e.g., the structure of rhythm perception vs. that of rhythm production, the role of stimulus-driven and short-term memory mediated bottom-up processes vs. long-term-memory-mediated top-down processes, near-universal perceptual and motoric functional capacities vs. effects of attention, familiarity, musical training and/or culture),
- Draw on different kinds of observables (physiological and neurophysiolgical measurements; overt behaviors such as finger-tapping; subject judgments such as pattern discriminations, recognitions, pattern matches; simulations of physical systems and neural networks)
- Explain these aspects in different terms (explanans, e.g., psychological representations and operations, neural information processing, signal analysis, correlation-processes, linear and nonlinear oscillators, dynamical systems, statistical inferences),
- Operate on different levels of description, e.g., high-level symbols-and-rules models vs. mid-level signal processing models vs. low-level, detailed biophysical, neuronal, and dynamical systems models.
- Are inspired by different physical, biological, psychological, neurophysiological, behavioral, mathematical, and musical ideas and examples, and therefore interpret their findings in very different ways
- Have different predictive and/or explanatory purposes, i.e., what constitutes a good explanation (e.g., precise predictions of specific phenomena vs. explanation of a wide array of phenomena).

Where does our RTN model fit within this bewildering array of convergent and divergent approaches? Theories that currently predominate are oscillatory dynamic attending and predictive coding models. The RTN model has many similarities to both, but also some significant differences. We will discuss them in turn.

### 8.8 RTNs and oscillator networks

First and foremost, the RTN model focuses wholly on rhythm *perception* rather than *production*. Most basic auditory percepts are, by and large, highly independent of motoric action. There is relative autonomy between systems that mediate perception and action. We disagree with pure, unidirectional motor theories of perception. Strong motor theories hold that there is no perception without motor system engagement, whereas weak motor theories hold that rhythm perception is mainly driven by motor systems. While we are sympathetic to weak enactivism, that the function of most brain activity is to guide external action so as to satisfy current goals, we are skeptical of strong enactivism (all neural activity is devoted to externalized action) and strong motor theories of perception (no perception without motoric activations, e.g., as in strong motor theories of speech perception). While this assertion may be defensible for some sensory modalities and attributes (e.g., touch, propriocention, balance and other vestibular attributes, external localizations), it is highly questionable for many “distal” senses such as the perception of auditory qualities (pitch, timbre, loudness), visual forms, and olfactory smells. In order to gain new information from environments, percepts must not be completely determined by internal processes, such as prior expectations or current states of cognition, emotion, or motivation.

That having been said, we recognize the bi-directional interplay between systems mediating perception and action. Rhythm perception and production involve the same temporal patternings of spikes, such that rhythmic patterns of neuronal activity produced in sensory areas could be directly coupled with motor areas to trigger motoric action. Conversely, patterns of movements (motor commands and proprioceptive feedback) can similarly interact to amplify incoming sensory patterns. For example, auditory neural signals can directly drive motor commands and temporal patterns of movement can weakly amplify similar patterns in auditory inputs. This works because both perceptual and motor representations are, at least in part, temporally coded. A significant part of the coordination of muscles in movement involves controlling the relative timings of different muscle groups (Merchant and Yarrow, 2016). The time pattern of a rhythm can be mapped onto these sets of motor commands.

### 8.9 Rhythm, pitch, oscillations, autocorrelations

Autocorrelation analysis of repetitive patterns produces many, if not most, of the same predictions that are made by oscillator models. These include multilevel, concurrent representations of all the different periodicities in rhythmic event patterns. Both running autocorrelation networks, such as the RTN model, and oscillator networks produce output waveforms that serve as predictions of the incoming event patterns. Both operate on temporally patterned inputs. Both can account for basic phenomena such as beat detection and filling in of missing beats. A main difference between them concerns the nature of their neural mechanisms. Oscillatory theories rely on oscillatory dynamics in individual neurons and neural populations, whereas autocorrelation models have relied on arrays of neural delays and coincidence detectors. Neural delays can be implemented through conduction delays, multisynaptic re-entrant delay paths, and rebound characteristics of individual neurons. Multitudes of neural delays, fast and slow, are evident at cortical levels. The slow neural delays are adequate to represent and process the slow delays between events in rhythm.

Rhythm and pitch have many similarities. The RTN model comes out of our previous work on the neural coding of pitch and autocorrelation-like neural representations based on phase-locked spike timings and all-order interspike intervals. Interspike interval patterns, as analyzed using autocorrelation, easily explain the pitch corresponding to the fundamental frequency (F0) of a repeating waveform pattern. From this time-domain vantage point, rhythm perception looks very similar to pitch perception, albeit on much slower timescales. F0s for musical pitches span ~25–4,000 Hz, whereas tempos for musical rhythms span ~30–240 bpm or ~0.5–4 Hz. This frequency range for rhythm perception corresponds to the conventional delta frequency band for brain waves (Buzsáki, 2006).

Despite their different timescales, both kinds of temporal patterns produce percepts related to the waveform repetition period, be it a higher-frequency acoustic waveform in the case of pitch or the period of repeating event onsets in the case of rhythm. For rhythm, this is the fundamental period of discrete events, such as pulses or note onsets, which can be regarded as an “event-F0.” Both pitch and rhythm patterns produce percepts at the fundamentals of their waveform pattern, even when the fundamental is “missing,” i.e., there is no energy at F0 at the fundamental frequency itself in the power spectrum. Listeners readily hear both “missing fundamentals” related to pitch and “missing event fundamentals” of repeating rhythmic event patterns.

At the level of the auditory nerve, pitch is temporally coded, by virtue of the phase-locking of spikes to the incoming acoustic waveform (after cochlear filtering), but the upper limit of obvious phase-locking declines down to a few hundred Hz as one ascends the auditory pathway to the auditory cortex.

In similar fashion, rhythm is temporally coded by virtue of phase locking to event onsets. Event timings are precisely represented with sub-millisecond precision in first spike times in response to their acoustic onsets. Stimulus-driven temporal patterns are widely observed in cortical stations and provide the basis of all neurocompuational models of rhythm. This begs the question of whether rhythm, like pitch, might both depend on distributions of interspike intervals, albeit on different timescales. The perceived repetition period (the beat) would correspond to the most common pattern of event-driven interspike intervals in cortical populations.

Networks of recurrent delay paths in timing nets and networks of linear and nonlinear oscillators have some deep similarities in that the delay loops and the oscillators exhibit resonances. Each RTN loop has a specific delay, such that when an incoming temporal pulse pattern repeats at that delay, it causes maximal pulse coincidences to occur, and with it also an increase in (synaptic) weighting. The RTN operation is a kind of running, unwindowed running autocorrelation-like process, with the absolute timing of the signal playing a role analogous to phase in Fourier analysis. In the case of oscillatory arrays, each oscillator has a preferred period, its reciprocal being its resonant frequency. It essentially acts like a bandpass filter. A simple linear harmonic oscillator has three parameters: resonant frequency, current phase, and amplitude.

So one can regard the RTN as implementing signal processing by passing rhythm pattern signals through banks of temporal autocorrelators whereas arrays of oscillators instead pass these signals through banks of linear and/or nonlinear filters. Nonlinear oscillators can be somewhat more complex than their linear counterparts, with non-sinusoidal (but usually biphasic, one peak and one trough) waveforms and quasi-periodic dynamical system phase trajectories.

Oscillators and delay loops differ, however, in the information that the individual oscillators and loops contain. Because of their tunings, each oscillatory element tracks only one periodicity, and consequently carries only a fragment of a whole, complex rhythmic pattern. No one element carries the whole pattern. This presents the problem of how the whole pattern is integrated.

In contrast to oscillators, each RTN delay loop can carry complex pulse patterns, i.e., the whole repeating rhythm pattern. One could therefore regard the RTN loops as complex pulse pattern oscillators with different periods. One could also, with difficulty and much added complexity, model behaviors of RTNs using dynamical systems.

Because of their focus on rhythm production (beat tracking), and not rhythm perception per se, it is unclear to us whether or how well the oscillator models can account for Gestaltist perceptual groupings, especially for complex, repeating, non-metrical patterns of events. One would guess that these would be regarded as quasi-periodic cycles within some high-dimensional dynamical systems phase space.

One might think that another major difference between the two kinds of networks, correlators vs. oscillators, would be that the correlation networks are driven by the external stimulus and self-organize according to its correlation structure (tabula rasa), whereas the oscillator network has a set of fixed endogenous resonances, perhaps heavily shaped by resonances in motor systems that are associated with bodily limbs and their movements (i.e., a landscape with hills and valleys).

Both theories agree that the rhythmic surface structure (event onsets) is available to both auditory and motor cortices. They both assume that the outputs of RTN- and oscillator-arrays would directly drive motor systems, so as to explain beat followings and rhythm productions. In order to predict these rhythm productions, RTNs would need to be expanded to include either neural timing nets or oscillatory motor networks for motor control. The RTN would output to the motor system volleys of spikes that follow the external rhythm weighted by RTN expectancies, such that rhythm *perception*, while it can be influenced by motor system resonances, it does not depend critically on them. In contrast, in neural resonance theory (Large et al., 2023; Harding et al., 2025), motor system resonances and their feedbacks to the auditory system are necessary for rhythm perception.

Finally, the two theories may differ in whether they implement linear or nonlinear processing. The RTNs also have nonlinear processes (coincidences, adaptive weighting). Neural resonance theory holds that nonlinear processes may be necessary for perception and production of the “missing” event-fundamental (“missing beat”). These periodicities are observed in auditory cortex along with phase-locking to individual event onsets and other major periodicities in the stimulus (Nozaradan et al., 2011; Fujioka et al., 2012; Nozaradan et al., 2012; Nozaradan, 2014; Tal et al., 2017). There are also nonlinearities that can be introduced by multiplicative neuronal mixing processes and cross-frequency oscillatory couplings (Buzsáki and Freeman, 2015; Luff et al., 2024; Baker and Cariani, 2025). Whether nonlinear interactions between oscillators in auditory and motor cortices is the only possible explanation (Large et al., 2023; Harding et al., 2025) remains to be seen. For example, if motor cortex has access to the surface structure of the repeating event pattern, then simply locking onto the same, most salient event in the repeating sequence will generate an isochronous output rhythm at the “missing” beat rate. Things may not be as complicated as they might seem.

### 8.10 Neural timing nets and predictive coding

We are highly sympathetic to the idea that brains are, in large part, anticipatory systems (Cariani, 2017). As with predictive coding, we believe that brains make predictions about the world in service of effective (goal-satisficing) behavior. Dynamic attending and neural resonance theories have also been applied to musical expectancies (Large and Kim, 2019) and explicitly related to predictive coding (Large et al., 2023; Harding et al., 2025). We regard the RTN model as a form of predictive coding whose expectancies are mediated by recent temporal context (a few seconds) that is complemented by another predictive coding process that is based on experienced event probabilities acquired in more distant pasts. This is consistent with predictive coding theory, as we understand it, in postulating different mechanisms for the roles of short-term and long-term memory (Friston, 2005; Koelsch et al., 2019).

As in current predictive coding models, when there is no longer temporal structure in the recent context or there are high levels of ambiguity due to low signal-to-noise ratios, RTN predictions revert to the probabilities of individual events and shorter event temporal sequences. In such circumstances, in the RTN model the signals coursing through longer delay loops are uncorrelated with incoming signals, and their contributions to network expectancies are minimal relative to shorter-term patterns (e.g., temporal interval pattern ngrams of shorter lengths). In predictive coding models that integrate expectancies based on short- and long-term information (Clark, 2013), the balance between predictions based on short- and long-term information shifts toward reliance on long-term prior probabilities. However, when there exists strong short-term temporal structure with low ambiguity, as with repeating rhythmic patterns in music, predictions based on that structure predominate. In any case, under conditions of predictive uncertainty, arguably the best policy is not to rely on long-term prior probabilities, but to gather more evidence, ideally also from additional observables.

We hesitate to speculate on how Friston’s energy minimization principle (Friston, 2005, 2010; Friston, 2019) would be related to our correlation-based predictions. It is unclear to us how the correlation-based predictive weightings of the RTN delay loops might resemble or differ from the short-term predictive coding theory of Friston. The RTN predictive model resembles linear prediction coding (LPC) formulations that use running short-time autocorrelations (Makhoul, 1975; Markel and Gray, 1976).

Some of the apparent differences between RTNs, oscillatory networks, and predictive coding might be due to the very different terms in which they are cast. It may be that the operations of the various models could be mapped onto each other.

In the end, differences between RTNs and oscillator networks may be simply that one is couched in terms of temporal codes and correlation operations, whereas the other is couched in terms of oscillators and dynamical systems. The RTN operates in the time domain with processes that resemble running, adaptive autocorrelations and comb filters, whereas the nonlinear oscillators operate in the frequency domain as banks of nonlinear filters. Both types of networks output temporally patterned waveforms rather than patterns of channel activations. It may be helpful to describe both approaches in terms of signal processing (signal waveforms, impulse and frequency responses, ringing durations, adaptive behaviors) so that more direct comparisons can be made.

Likewise, the differences between RTNs and predictive coding may be largely differences between predictions couched in terms of correlations and those couched in terms of predictive error minimizations.

## 9 Future directions: empirical testing, theoretical work, possible practical implications

What theoretical and empirical work needs to be done to develop the RTN model? Theoretical work could include simulations-based demonstrations that show that STDP-facilitated multisynaptic delay paths can support provisionally-stable synfire cycles and temporal memory traces. How might running, correlation-like operations be realized via biophysically-realistic STDP processes? Clearer understanding of logical equivalences and differences between correlation-based short-time prediction operations, oscillatory networks, and predictive coding processes would be desirable. Can selective coupling of rebound and/or of ringing neural oscillators play the same roles as delay-paths?

A deeper problem involves explaining rhythmic pattern invariance w.r.t. changes in tempo. For time-domain representations this requires a time scaling operation (time dilation, compression). How does the MMN change when the same rhythmic pattern is presented at different tempos?

Model refinements could include better grounding of model assumptions regarding STDP-biophysics and neural coding of event attributes. In the longer term, RTNs could be applied to attempt to predict observed MMN-like neural responses by situating the delays and coincidence operations in specific sets of neural populations.

Experimental work might include testing predictions of the RTN model for missing pulse phenomena (Tal et al., 2017). Rhythm-tagged stimuli in multiple modalities (auditory, visual, vibrotactile) could be used to better understand stimulus-driven and stimulus-induced rhythmic patterns in echoic and working memory. Neural mechanisms underlying rhythm assimilation in neurons (Morrell, 1967) ought to be further examined.

Because we believe that precise spike timing and temporal processing may be much more important to major brain functions than has been commonly assumed (Cariani and Baker, 2022; Baker and Cariani, 2025; Cariani and Baker, 2025), spike timing disruptions could well be underlying causes of various neurological and psychiatric dysfunctions, e.g.(Andrade-Talavera et al., 2023). However, time domain neural signal processing models remain to be further refined and better grounded in specific neural populations and structures. Once these are achieved, more specific hypotheses can then be formulated and their relevance to disease states can be assessed.

What might be some possible technological implications? Neural timing networks could prove useful for new kinds of artificial neural networks that deal with temporal sequence predictions. Problems of discriminating, recognizing, representing, and generalizing from temporal patterns and sequences were recognized early on in the development of neural networks (Rosenblatt, 1958, 1962).

Time ought to be incorporated in neural networks as a fundamental dimension. New kinds of time-domain neural networks would incorporate time as a basic parameter. They could incorporate temporally-coded signals alongside other signal types. We envision them as massively parallel spiking neural networks consisting of rich sets of delays, adaptive coincidence-dependent synaptic weightings, and coincidence detectors. Hybrid systems made up of time-domain and existing networks are also feasible. It is our belief that that there are many powerful new principles for designing artificial information processing systems that can yet be learned from studying the structures and functions of nervous systems that Nature has given us.

## 10 Conclusion

Despite its simple structure, as a demonstration-of-concept, the recurrent neural timing net (RTN) model does appear to behave in a manner that is qualitatively similar to the perception of repeating rhythmic patterns and of MMN-like neural responses. It is a preliminary mode that bears further investigation, refinement, and empirical testing. With appropriate additions, it could be modified to attempt to predict MMN-like responses.

What is novel about the (RTN) model? First, the model explicitly uses temporally-coded input signals, i.e., stimulus-driven temporal patterns of neural spike volleys associated with the onsets of sensory events. Although this is implicitly assumed in models of rhythm perception, it is rarely made clear. In contrast to anticipatory models based on previously acquired experiences (training, enculturation), the model demonstrates how rhythmic pattern expectancies can also be based on recent temporal pattern (auto) correlations in the incoming acoustic signal. It outlines how a network of delay loops (composed of multisynaptic delay paths) and coincidence detectors can compute a running, windowless autocorrelation-like transform.

The model uses temporal memory traces in reverberating circuits. To our knowledge, the notion of an iconic temporal memory trace and delay-based, tape-recorder-like short-term memory is absent from the current discussions of rhythm perception. Although such a “cyclochronism” was proposed by the Russian psychologist Popol almost 80 years ago, the idea was always obscure, and by now it has been all but forgotten. This model revives that idea.

The RTN model uses correlation-based predictions rather than Bayesian estimation. The adaptive weighting of delay loop predictions based on short-term running predictive success (STDP-like coincidence based mechanism) appears to be an effective strategy for situations where there is a high degree of repetition, such as in music perception.

## Data Availability

The original contributions presented in the study are included in the article/supplementary material, further inquiries can be directed to the corresponding author.

## Appendix

Pseudocode for the simple algorithm presented in this paper. The algorithm was implemented in Matlab.

1. Choose a sampling rate and time base.
  For only event onsets, sampling period of ~50 ms is sufficient.
  For representing auditory features of events, higher temporal resolution is needed.
2. Choose a temporal coding scheme based on binary-valued pulse patterns (0, 1).
  Single pulses for each event vs. finer, complex pulse patterns encoding attributes.
3. X(t) is the input signal.
4. Set up series of delay loops_i_ with delay tau and delay matrix of X(t, t-tau).
5. For each delay loop_i_ at time t, compute the Pearson correlation coefficient (PCC) between the signal of the last period tau and that of period preceding it. Alternately, compute this value over some fixed time window.
6. The prediction at time t of each delay loop tau_i_ is the value of X (0 or 1) at time t-tau_i_.
7. For each timestep, sum the PCCs of loops predicting 0 (SPCC_0_) and those predicting 1, (SPCC_1_).
8. Each loop prediction will be weighted by its correlation value over the last two periods.
9. The prediction of the whole array is the value (0, 1) of whichever SPCC was greatest.
10. Compare the array prediction with the actual value of the incoming signal at each time t
11. If the two values are the same, the array prediction was correct (error magnitude = 0).
12. If they are different, the normalized magnitude of the error = abs(SPCC_0_ − SPCC_1_)/(#loops).
13. Plot the input signal, the array predictions, prediction errors and their normalized magnitudes.
